# Oncofertility case report: live birth 10 years after oocyte in vitro maturation and zygote cryopreservation

**DOI:** 10.1007/s10815-020-01984-3

**Published:** 2020-10-28

**Authors:** P. Rodrigues, M. Marques, S. Pimentel, M. Rato, P. Carvalho, S. C. Correia, N. Mendes, H. Amaral, J. P. Fernandes, M. J. Carvalho, C. E. Plancha

**Affiliations:** 1 Centro Médico de Assistência à Reprodução - CEMEARE, Lisbon, Portugal; 2 Escola de Psicologia e Ciências da Vida, Universidade Lusófona de Humanidade e Tecnologia de Lisboa, Lisbon, Portugal; 3 Hospital CUF Descobertas Lisboa, Lisbon, Portugal; 4 Inst. Histologia e Biol. Desenvolvimento, Faculdade de Medicina, Universidade de Lisboa, Lisbon, Portugal

**Keywords:** Oocyte in vitro maturation, Gonadotoxicity, Fertility preservation, Controlled ovarian stimulation, Oncofertility

## Abstract

**Purpose:**

This study aims to report a case of urgent fertility preservation in an oncological patient with collection of immature oocytes in the absence of ovarian stimulation that, through in vitro maturation (IVM), followed by ICSI and cryopreservation of zygotes resulted, 10 years later, in the live birth of a healthy baby.

**Methods:**

In September 2008, our clinic performed IVM in a 32-year-old woman diagnosed with a ductal invasive carcinoma with positive estradiol receptors, negative progesterone receptors and positive human epidermal growth factor receptor 2. The retrieval of immature oocytes was performed in the absence of ovarian stimulation after a simple mastectomy and prior to any chemotherapy treatment. The compact cumulus-oocyte complexes (COCs) collected were placed in Lag medium for 2 h, followed by incubation in IVM medium, supplemented with heat inactivated patient serum, recombinant FSH, and recombinant LH. After 30 h in culture, cumulus cells were removed, the metaphase II oocytes were microinjected, and the zygotes obtained were cryopreserved. In 2017, the zygotes were thawed and cultured until day 3. One embryo was transferred and the other cryopreserved.

**Results:**

Four compact COCs were collected and subjected to IVM. Two oocytes reached metaphase II and were microinjected. Two zygotes were obtained and were cryopreserved at the two pronuclear stage. Approximately 9 years later, the two zygotes were thawed and cultured until day 3. An embryo with 10 cells was transferred and implanted, resulting in the birth of a healthy baby.

**Conclusions:**

In cases where urgency to start adjuvant therapy requires immediate oocyte collection, IVM may be the only option to obtain fully competent mature oocytes allowing for effective preservation of the reproductive potential.

## Introduction

Long-term life expectancy and survival rates of cancer patients have continuously increased, due to both diagnosis and treatment improvements. However, the toxicity of the treatments involved in oncotherapy can be detrimental to the gonads, shortening or even eliminating the reproductive potential in these patients [1–3].

Although the incidence of cancer is higher in patients over 50 years old, there is a growing number of diagnoses in younger patients. In particular, breast cancer accounts for one-third of cancer diagnosis in reproductive-aged women [4, 5]. As a result, there is an increasing number of these women seeking to preserve their reproductive potential. Depending on the specific diagnosis, patient’s age, and emergency of treatment initiation, it can be suggested oocyte, embryo, or ovarian tissue cryopreservation [6].

Oocyte in vitro maturation (IVM) is an alternative method that does not rely on COS protocols and so does not require delays in gonadotoxic treatment initiation [7, 8]. IVM allows for immature oocyte collection and subsequent oocyte culture independent of the specific day of the menstrual cycle. The procedure from immature oocyte collection to mature oocyte cryopreservation takes less than 48 h [9]. This technique has been proven as a viable option for preservation of the reproductive potential for oncological patients either through mature oocyte or embryo cryopreservation, particularly in urgent or when COS is not an option [10–13].

There are only a few live birth reports of oncological patients after IVM, however with limited success rates considering the number of cases included in the studies [10, 12]. Here we report one successful clinical case although with few oocytes. In this report, clinical contraindication for COS and requirement for urgent cytotoxic treatment prompted for IVM application. Following couple’s request, the mature oocytes were microinjected, and the resulting zygotes were cryopreserved. Nine years later, the zygotes were thawed and allowed to develop further. A single embryo transfer was performed at day 3, resulting in a pregnancy. Both pregnancy and delivery of a healthy baby boy, by caesarean section, were completed without complications. The other embryo remains cryopreserved.

## Material and methods

### Oocyte retrieval

Transvaginal follicular puncture was performed on 6th day of menstrual cycle, during the follicular phase, under mild anesthesia and without any ovarian stimulation. The cumulus-oocyte complexes (COCs) were collected with an aspiration pressure of 80–90 mmHg, and an aspiration needle (16-gauge/35 cm, Cook) with a single lumen into a centrifuge tube with 5 ml of Flushing media with heparin (MediCult, Origio, Denmark). The follicular fluid was filtered with a 70 μm cell strainer (Corning - BD Falcon, NY, USA). The collected COC’s were washed with Flushing media (MediCult, Origio, Denmark).

### IVM, fertilization, and embryo culture

IVM medium was prepared according to the manufacturer’s instructions (Origio, Denmark). Briefly, the media was pre-equilibrated overnight at 37 °C and 5% CO_2_. COCs were placed in a 4-well dish with the 2 superior wells with 500 μl of Lag medium (MediCult IVM System, Origio, Denmark) covered with paraffin (MediCult, Origio, Denmark), for 2 h. IVM medium (MediCult IVM System, CooperSurgical, Denmark) was supplemented with heat inactivated patient serum (20%), recombinant FSH (at final concentration of 1 IU/μl) (Gonal F®, Merck Serono), and recombinant LH (at final concentration of 1 IU/μl) (Luveris®, Merck Serono). The COCs were transferred to the supplemented IVM medium in the 2 inferior wells of the 4-well dish, also covered with paraffin and incubated for 30 h. Denudation of the COC’s was done both mechanically and enzymatically (Synvitro Hyadase, MediCult, Origio, Denmark), using a 170-nm denudation pipette, and ICSI was performed to the mature oocytes, incubated in ISM1 (MediCult, Origio, Denmark) covered with paraffin, at 37 °C and 5% CO_2_. Semen sample was prepared using three density gradients (90%-70%-50% PureSperm-100, Nidacon, diluted in SPM, Origio, Denmark), followed by two washes in SPM (Origio, Denmark), and resuspended in SPM.

### Cryopreservation and thawing

The zygotes obtained were cryopreserved following the slow freezing protocol, according to the manufacturer’s instructions (MediCult Freezing Pack, Origio, Denmark). Thawing of the zygotes in 2017 followed the slow thawing protocol (Origio Embryo Thawing Pack, Origio, Denmark) and cultured until day 3 in Cleav (Sequential Cleav, Origio, Denmark). One embryo was transferred and the other was vitrified (Irvine Freezing Kit, FujiFilm, CA, USA).

### Embryo transfer

**Fig. 1:**
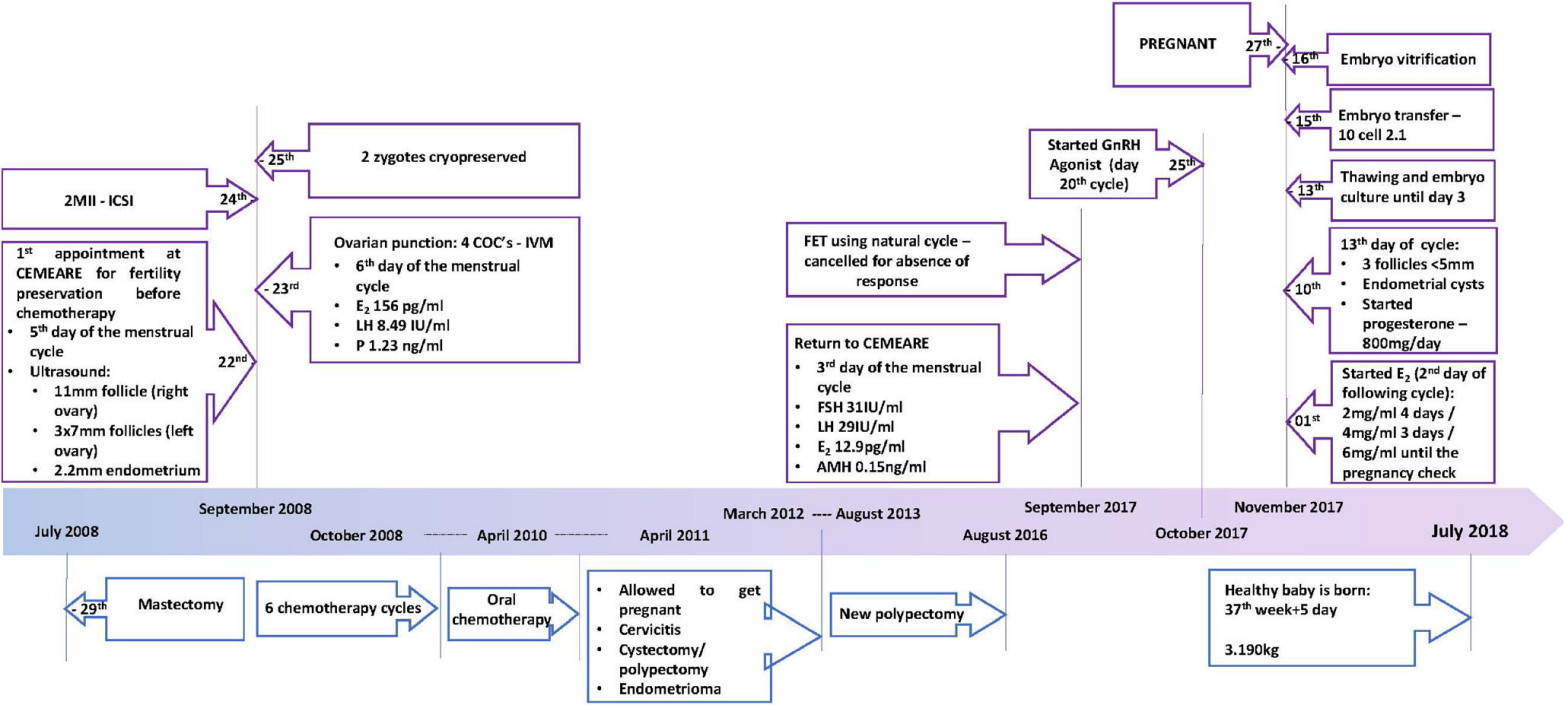
Resume of clinical medical history and procedures

The first approach for endometrial preparation was a natural cycle, which was canceled due to the absence of response (Fig. 1).

A hormonal replacement treatment was started with a GnRH agonist (Ago) Decapeptyl 3.75 mg on the 20th day of the previous cycle and endometrial preparation with E_2_—Estrofem daily started on the 2nd day of the following cycle. E_2_ initiated with 2 mg/day and progressively went up to 6 mg/day. On the 13th day of the cycle, the patient blood tests showed E_2_ 348pg/ml and P 0.3 ng/ml. The ultrasound revealed an endometrium with 8.8 mm and presence of an endometrial cyst on the right ovary. Luteal support (Progeffik) 800 mg/day vaginally started on the 13th day of the cycle.

The embryo transfer was ultrasound guided, on the 3rd day of progesterone supplementation (Progeffik). Transfer was performed with a COOK Sydney IVF Embryo Transfer Set (Cook Medical, USA) catheter.

## Results

### IVM, ICSI, and zygote cryopreservation

A 32-year-old woman with breast cancer requested to preserve her reproductive potential prior and to chemotherapy and after having simple mastectomy and sentinel lymph node removed. The histological analysis post-surgery revealed an invasive hormone-dependent ductal carcinoma, grade II—pT1a pN0(sn), human epidermal growth factor receptor 2 (HER2) positive, 25% positive for estradiol receptors (Er), and negative for progesterone receptors (Pr). It was proposed IVM without COS or any oocyte maturation trigger. The patient was informed of the technique limitations and that embryo transfer should only be performed after ending chemotherapy, when the oncology group considered it safe.

The first ultrasound observation was on the 5th day of spontaneous menstrual cycle of the patient, showing 3 follicles of 7 mm (left ovary), one follicle of 11 mm (right ovary), and a 2.2 mm endometrium. The blood tests depicted: estradiol (E_2_) 156 pg/ml, LH 8.49UI/ml, and progesterone (P) 1.23 ng/ml (Fig. 1).

Semen analysis revealed an OligoAstenoTeratozoospermia, with 14 × 10^6^/mL sperm concentration, 3% of morphologically normal forms, and 5% of progressive sperm motility, a clear indication to ICSI in our clinic.

**Fig. 2:**
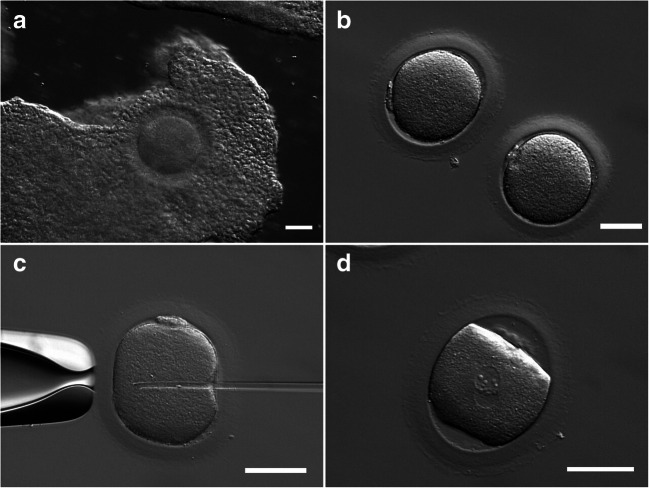
COCs placed in IVM (**a**), two MII oocytes recovered 30 h afterwards (**b**), were microinjected (**c**), and the following day zygotes were cryopreserved (**d**). Scale bars 50 μm

Transvaginal follicular aspiration was performed, on the following day (6th day) to four follicles, three of them under 10 mm and one with 14 mm, in the absence of ovarian stimulation. Four COCs were collected and IVM was performed. The COCs presented compact corona cells surrounding the oocytes, as expected (Figs. 1 and 2a). Thirty hours post IVM culture, the 2 mature oocytes obtained were microinjected (the other two oocytes remained at the germinal vesical stage) (Fig. 2b–c). Complying with the couple request, based on ethical values and pragmatic attitude, the fertilized oocytes were cryopreserved at the zygotic stage, before pronuclear disappearance (Fig. 2d).

### Chemotherapy and spontaneous pregnancy attempts

After oocyte retrieval, the patient immediately started chemotherapy (October 2008) ending on April 2010 (6 consecutive cycles followed by 1 year of treatment), under supervision and guidance of Oncological Consultation at CUF Hospital and the Portuguese Institute of Oncology (IPO). In March 2012, the patient was considered in complete remission from the oncological disease and allowed to become pregnant.

In August 2012, the patient was diagnosed with an endometrial cyst in the left ovary, which was kept under vigilance for 1 year. In August 2013, through laparoscopy and ressectoscopy, at the IPO, the patient was subjected to a cystectomy on the left ovary (due to endometrioma) and to endometrial polypectomy (Fig. 1). In 2016, hysteroscopy with polypectomy was repeated (Fig. 1).

### Use of zygotes after IVM fertility preservation

In September 2017, the couple returned to CEMEARE, after almost 5 years of trying to get pregnant naturally. Even though the woman was still cycling, with irregular cycles, the anti-Mullerian hormone (AMH) was low: 0.15 ng/ml (Fig. 1). Other blood analysis on the 3rd day of the cycle showed FSH of 31UI/ml, LH 28UI/ml, and E_2_ 12.9 pg/ml (Fig. 1). The patient showed to be immune to rubella, varicella, and cytomegalovirus, with no immunity to toxoplasmosis. All obligatory serology testing (HIV, HBV, HCV, syphilis) was negative. A frozen embryo transfer (FET) in an estrogen and progesterone supplemented cycle was programmed.

**Fig. 3:**
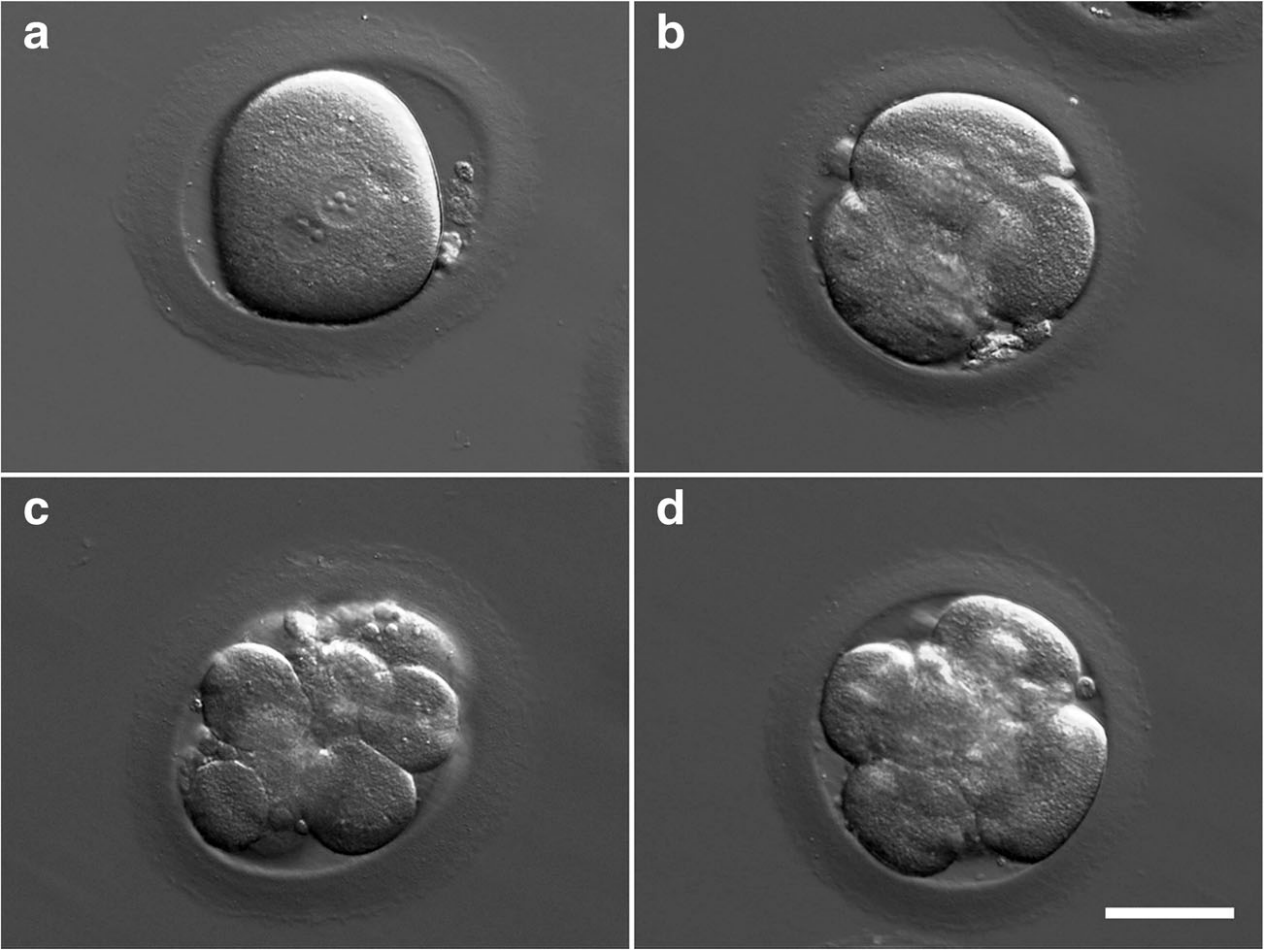
Embryo development post-thawing: (**a**) zygote post-thawing; (**b**) 4-cell embryo on day 2 of development; (**c**) 10-cell embryo on day 3, transferred embryo; and (**d**) 6-cell embryo on day 3, vitrified embryo on day 4. Scale bar 50 μm

The patient, 42 years old, was treated with agonist (Ago), E_2_ and P (see materials and methods) for endometrium preparation. In the laboratory, the zygotes were thawed and left in culture until day 3 (Figs. 1 and 3). Complying with the clinician advice, the couple decided to transfer a single embryo. Given the risk of low success reaching a blastocyst stage with only one embryo, it was decided by the clinical embryologist team that the transfer should be performed at day 3.Although both embryos exhibited a regular development, the selection was easy since one embryo exhibited better quality, with 10 uneven cells and less than 10% of fragmentation (Fig. 3c), while the other embryo exhibited 6 uneven cells and around 10% of fragmentation (lower quality). The remaining embryo presenting with less cells, was left in culture one more day, before cryopreservation, in order to exclude developmental arrest. On day 4, this embryo presented 9 uneven cells and was vitrified (Fig. 3d).

Twelve days after transfer, the βhCG result was 233 IU (Fig. 1). Four weeks after transfer, ultrasound revealed one gestational sac in uterus and one embryo with cardiac movements corresponding to an embryo aged 6 weeks and 1 day. The progesterone (800 mg/day) and E_2_ (6mg/day) vaginal support was monitored until the 12th week of gestation.

First trimester combined screening (maternal blood and ultrasound evaluation of the fetus) was performed on the 13th week of gestation. The patient was followed in CUF Descobertas Hospital during pregnancy where a caesarean delivery took place at 37th week and 5 days. A healthy baby boy, weighing 3.190 kg, with Apgar scores 10-10, was born on July 2018, with no apparent abnormalities (Fig. 1).

At 1 year of age, normal development was confirmed by the pediatrician.

## Discussion

Here we report the birth of a healthy baby 10 years after IVM and cryopreservation of zygotes. The patient contacted the reproductive clinic post-mastectomy and prior chemotherapy for preservation of her reproductive potential. Even though the ovarian stimulation might had been possible in this particular case, the reproductive team at CEMEARE and the oncological group at CUF Hospital, 10 years ago, preferred not to induce stimulation also due to the urgent need of cytostatic treatment initiation, which prompted for application of the IVM procedure.

IVM is mainly indicated in polycystic ovarian syndrome (PCOS) patients, with high antral follicle counts, trying to decrease their risk of developing ovarian hyperstimulation syndrome induced by different ovarian stimulation protocols [14, 15]. IVM can also be an option for a variety of other conditions like poor responders [16], preservation of the reproductive potential on oncological patients [17], premature ovarian failure, autoimmune diseases, and others [10, 11, 18].

Breast cancer patients with contraindication to undergo ovarian stimulation widely benefit from using IVM and cryopreservation of mature oocytes and embryos [5, 18, 19]. Previously, the major obstacles for cancer patients wishing to preserve their reproductive potential were the urgency of cytotoxic treatment or the impossibility of ovarian stimulation. IVM allowed to overcome it without delaying treatment, as was demonstrated by Kitano and colleagues [1]. Thus, for cancer patients, IVM may represent an important source of mature oocytes for preservation of their reproductive potential [11].

The first report of a baby born from IVM was on 1991, from donor oocytes, in Korea [20]. Three years later, in Australia, Trounson and colleagues reported the first mother’s own immature oocytes IVM baby [14]. Since then, worldwide there have been approximately 5000 birth reports from IVM [18] with similar obstetric and neonatal outcomes to COS [21]. However, IVM maintains a variable perception of low fertility and pregnancy rates [15, 22–24].

Significantly, in this case, the oncological patient returned to our clinic 9 years after zygote cryopreservation, at 42 years of age, still cycling, and after 5 years attempting to naturally conceive. Although after fertility preservation and oncology treatments the first option of most patients is to naturally conceive [25], the female age and the low AMH level (0.15 μg/l) justified the use of assisted reproductive techniques (ART). This case resulted in a live birth without complications, as similarly reported by Garrido-Marrín and colleagues [4]. In both cases, the patients returned to the clinic post-chemotherapy and with over 40 years of age [4]. Healthy live births were also achieved in a combination of IVM and oocyte cryopreservation, despite lower efficiency when compared with using fresh IVM oocytes [3, 12]. There is another report of fertility preservation after IVM, also without ovarian stimulation, but the patient has not yet returned for embryo transfer [19]. Recently, Creux and colleagues [10] reported a live birth after an IVM cycle with fertilization and cryopreservation of embryos, in a cancer patient. Another recent study, reported a birth after IVM embryo cryopreservation from a cancer patient with PCOS [12]. Although these recent studies are compilations of several cases, a closer look at the numbers seem to reveal a limited success rate with the use of embryos cryopresed after the IVM procedure [10, 12]. This further emphasizes the relevance of this case report and aims to reinforce the idea that oocytes obtained after IVM may be relevant in fertility preservation, even at low numbers.

The extra training and workload both for the embryologists and gynecologists inherent to IVM protocols may be additional factors decreasing choice of this technique over antagonist protocols of COS [23]. However, there are now clear improvements in the culture protocols that may enhance the results of IVM [23]. Additionally, in some cases, like the one we report here, where urgency to start adjuvant therapy required immediate oocyte collection, IVM may be the only opportunity to obtain fully competent mature oocytes for reproductive potential preservation. Therefore, we believe that this technique should be recommended for reproductive potential preservation in oncological patients, whenever ovarian stimulation is not an option.

## References

[1] Kitano A (2019). Factors associated with treatment delay in women with primary breast cancer who were referred to reproductive specialists. ESMO Open.

[2] Tammiste T (2019). A case report and follow-up of the first live birth after heterotopic transplantation of cryopreserved ovarian tissue in Eastern Europe. BMC Womens Health.

[3] Son W-Y, Henderson S, Cohen Y, Dahan M, Buckett W (2019). Immature oocyte for fertility preservation. Front Endocrinol (Lausanne).

[4] Garrido-Marín M, Argacha PM, Fernández L, Molfino F, Martínez-Soler F, Tortosa A, Gimenez-Bonafé P (2019). Full-term pregnancy in breast cancer survivor with fertility preservation: A case report and review of literature. World J Clin Cases.

[5] Grynberg M, Poulain M, Le Parco S, Sifer C, Fanchin R, Frydman N (2016). Similar in vitro maturation rates of oocytes retrieved during the follicular or luteal phase offer flexible options for urgent fertility preservation in breast cancer patients. Hum Reprod.

[6] Oktay K, Harvey BE, Partridge AH, Quinn GP, Reinecke J, Taylor HS, Wallace WH, Wang ET, Loren AW (2018). Fertility preservation in patients with cancer: ASCO clinical practice guideline update. J Clin Oncol.

[7] Huang JYJ, Chian RC, Gilbert L, Fleiszer D, Holzer H, Dermitas E, Elizur SE, Gidoni Y, Levin D, Son WY, Tan SL (2010). Retrieval of immature oocytes from unstimulated ovaries followed by in vitro maturation and vitrification: a novel strategy of fertility preservation for breast cancer patients. Am J Surg.

[8] Shalom-Paz E, Almog B, Shehata F, Huang J, Holzer H, Chian RC, Son WY, Tan SL (2010). Fertility preservation for breast-cancer patients using IVM followed by oocyte or embryo vitrification. Reprod BioMed Online.

[9] Creux H, Monnier P, Son WY, Tulandi T, Buckett W (2017). Immature oocyte retrieval and in vitro oocyte maturation at different phases of the menstrual cycle in women with cancer who require urgent gonadotoxic treatment. Fertil Steril.

[10] Creux H, Monnier P, Son WY, Buckett W (2018). Thirteen years’ experience in fertility preservation for cancer patients after in vitro fertilization and in vitro maturation treatments. J Assist Reprod Genet.

[11] Oktay K, Buyuk E, Rodriguez-Wallberg KA, Sahin G (2010). In vitro maturation improves oocyte or embryo cryopreservation outcome in breast cancer patients undergoing ovarian stimulation for fertility preservation. Reprod BioMed Online.

[12] Kedem A, Yerushalm GM, Ranani H, Orvieto R, Hourvitz A, Meirow D (2018). Outcome of immature oocytes collection of 119 cancer patients during ovarian tissue harvesting for fertility preservation. J Assist Reprod Genet.

[13] Segers I, Barghil E, Mateizell I, Moer EV, Schots R, Verheyen G, et al. Live births following fertility preservation using in-vitro maturation of ovarian tissue oocytes. *Hum Reprod*. 2020:1–13. 10.1093/humrep/deaa175. 10.1093/humrep/deaa175 32829388

[14] Trounson A, Wood C, Kausche A (1994). In vitro maturation and the fertilization and developmental competence of oocytes recovered from untreated polycystic ovarian patients. Fertil Steril.

[15] Walls ML, Hunter T, Ryan JP, Keelan JA, Nathan E, Hart RJ (2015). In vitro maturation as an alternative to standard in vitro fertilization for patients diagnosed with polycystic ovaries: A comparative analysis of fresh, frozen and cumulative cycle outcomes. Hum Reprod.

[16] Li J, Xu Y, Zhou G, Guo J, Xin N (2011). Natural cycle IVF/IVM may be more desirable for poor responder patients after failure of stimulated cycles. J Assist Reprod Genet.

[17] Loren AW, Mangu PB, Beck LN, Brennan L, Magdalinski AJ, Partridge AH, Quinn G, Wallace WH, Oktay K, American Society of Clinical Oncology (2013). Fertility preservation for patients with cancer: American Society of Clinical Oncology clinical practice guideline update. J Clin Oncol.

[18] Hatırnaz Ş, Ata B, Saynur Hatırnaz E, Dahan MH, Tannus S, Tan J, Tan SL (2018). Oocyte in vitro maturation: a systematic review. Turk Jinekoloji ve Obstet Dern Derg.

[19] Sabine R, Stephanie H, Ariane G, Thomas S (2019). Successful in vitro maturation for urgent fertility preservation despite hormonal contraception by continuous progestin application. Gynecol Endocrinol.

[20] Cha KY, Koo JJ, Ko JJ, Choi DH, Han SY, Yoon TK (2016). Pregnancy after in vitro fertilization of human follicular oocytes collected from nonstimulated cycles, their culture in vitro and their transfer in a donor oocyte program**Prize paper, presented at the 45th Annual Meeting of The American Fertility Societ. Fertil Steril.

[21] Mostinckx L (2019). Obstetric and neonatal outcome of ART in patients with polycystic ovary syndrome: IVM of oocytes versus controlled ovarian stimulation. Hum Reprod.

[22] Herta AC, Lolicato F, Smitz JEJ (2018). In vitro follicle culture in the context of IVF. Reproduction.

[23] Sánchez F, Lolicato F, Romero S, de Vos M, van Ranst H, Verheyen G, Anckaert E, Smitz JEJ (2017). An improved IVM method for cumulus-oocyte complexes from small follicles in polycystic ovary syndrome patients enhances oocyte competence and embryo yield. Hum Reprod.

[24] Child SL, Tim J, Abdul-Jailil AK, Gulekli B, Tan (2001). In vitro maturation and fertilization of oocytes from unstimulated normal ovaries, polycystic ovaries, and women with polycystic ovary syndrome. Fertil Steril.

[25] Schmidt KT, Nyboe Andersen A, Greve T, Ernst E, Loft A, Yding Andersen C (2013). Fertility in cancer patients after cryopreservation of one ovary. Reprod BioMed Online.

